# PLGA-Carbon Nanotube Conjugates for Intercellular Delivery of Caspase-3 into Osteosarcoma Cells

**DOI:** 10.1371/journal.pone.0081947

**Published:** 2013-12-03

**Authors:** Qingsu Cheng, Marc-Olivier Blais, Greg Harris, Ehsan Jabbarzadeh

**Affiliations:** 1 Department of Biomedical Engineering, University of South Carolina, Columbia, South Carolina, United States of America; 2 Department of Chemical Engineering, University of South Carolina, Columbia, South Carolina, United States of America; 3 Department of Orthopaedic Surgery, University of South Carolina, Columbia, South Carolina, United States of America; The Ohio State University, United States of America

## Abstract

Cancer has arisen to be of the most prominent health care issues across the world in recent years. Doctors have used physiological intervention as well as chemical and radioactive therapeutics to treat cancer thus far. As an alternative to current methods, gene delivery systems with high efficiency, specificity, and safety that can reduce side effects such as necrosis of tissue are under development. Although viral vectors are highly efficient, concerns have arisen from the fact that viral vectors are sourced from lethal diseases. With this in mind, rod shaped nano-materials such as carbon nanotubes (CNTs) have become an attractive option for drug delivery due to the enhanced permeability and retention effect in tumors as well as the ability to penetrate the cell membrane. Here, we successfully engineered poly (lactic-co-glycolic) (PLGA) functionalized CNTs to reduce toxicity concerns, provide attachment sites for pro-apoptotic protein caspase-3 (CP3), and tune the temporal release profile of CP3 within bone cancer cells. Our results showed that CP3 was able to attach to functionalized CNTs, forming CNT-PLGA-CP3 conjugates. We show this conjugate can efficiently transduce cells at dosages as low as 0.05 μg/ml and suppress cell proliferation up to a week with no further treatments. These results are essential to showing the capabilities of PLGA functionalized CNTs as a non-viral vector gene delivery technique to tune cell fate.

## Introduction

Cancer is a principal concern in today’s health care treatment with more than 10 million cases each year[1]. Oncologists generally utilize physiological intervention as well as chemical and radioactive therapeutics to treat different forms of cancer. The drawbacks to the current therapeutics are the removal or necrosis of healthy tissue in addition to the tumor tissue. These side effects are one of the major concerns for traditional cancer treatments. To this end, targeted drug delivery has paved another avenue in potential cancer therapy in recent years. The Food and Drug Administration (FDA) approved clinical gene therapy in the 1990s[2] and in experimental and clinical stages has since demonstrated the promise of drug delivery in curing human diseases. Retrovirus[3], adenovirus[4] and adeno-associate[5] viral vectors are the major types of gene vectors used in research today. Although viral vectors are efficient in both delivery and transduction of cells, there are concerns that these viruses are sourced from lethal diseases such as human immunodeficiency virus (HIV) and human T-cell lymphotropic virus (HTLV). A safe, inexpensive, and effective vector has yet to be fabricated for use in gene therapy. Therefore, developing these safe and efficient delivery systems in a controlled manner is a major focus and challenge in research[6]. 

The enhanced permeability and retention (EPR) effect, which is inherent to tumor biology, can allow nano-sized drug carriers to accumulate, retain, and release drugs due to the fact that tumors have leaky blood vessels and poor lymphatic drainage[7]. In further researching nano-sized drug carriers, scientists have begun widening the window into the dark room of cancer therapy. The use of nano-sized drug carriers has a few key advantages including the protection of delicate drugs, enhanced absorption in selective tissues, controlled drug distribution profile, and enhanced intracellular penetration[8]. Thus, researchers have taken initiative and developed several methods to fabricate nano-sized drug delivery carriers.

Polymers and lipids have garnered the most attention thus far as materials for drug delivery. Synthetic polymers such as poly (ethylene glycol) (PEG)[9], poly (lactic acid) (PLA)[10] and PLGA[11] as well as natural polymers such as chitosan[12], collagen[13], gelatin[14] or lipids[15] can be fabricated as nanoparticles, liposomes, and micelles to deliver molecules by either chemical modification or physical absorption. Drugs are able to be released in a controlled manner due to either surface and bulk degradation or phase transition principles. However, there are concerns due to immune responses dealing with the heterogeneity of the materials used as well as the low transfection efficiency and specificity. In noting this, we sought to develop a highly efficient non-viral vector drug delivery system utilizing CNTs.

 CNTs[16], silicon nanowires[17], gallium nanotubes[18], boron nitride nanotubes[19], titanium oxide nanotubes[20] and zinc oxide nano-rods[21] have received an overwhelming amount of support and enthusiasm in biomedical research. CNTs are made of a layer of graphene[22] and have been used to shuttle several different biological molecules, ranging from small drug molecules[23] to biomacromolecules such as proteins[24], DNA[25,26] and RNA[27] into different types of cells via endocytosis efficiently[28]. Ricin A is an example that has been delivered into numerous cell lines through conjugation of CNTs intended to induce cell death[29]. In addition to cell proliferation inhibition and induction of apoptosis, DNA plasmids were also delivered into cells and enhanced desired gene expression. CNTs are able to achieve this penetration of the cells and present the opportunity to transport biological cargo across the cell membrane due to an extremely high aspect ratio. However, health concerns have hampered the practical application of using inorganic CNTs in biological applications thus far. Toxicity issues still raise doubts about the practical applications of CNTs due to the intrinsic toxicity caused by their high surface area and hydrophobicity[30]. However, toxicity concerns are lessened in cancer therapy due to the EPR effect which accumulates nanoparticles in only tumor tissue limiting the potential toxicity of CNTs to strictly the cancer cells and the fact that CNTs at small amounts or functionalized with other molecules have shown to evoke minimum toxicity to normal cell lines [31,32]. Therefore, functionalization of CNTs can prove to be a successful path to protein and gene therapy. For instance, Liu et al. have been able to successfully deliver siRNA with phospholipid PEG (PL-PEG) functionalized CNTs into tumor cells and tissue to inhibit tumor cell proliferation, tissue ingrowth, and CXCR4 expressions both *ex vivo* and *in vivo*[33–35]. siRNA was released by breaking the S-S bond, which attached the siRNA to PL-PEG. Other pristine CNT based delivery vectors are able to rely on diffusion to unload biological cargoes[24–27]. Although the cellular uptake mechanism may differ depending on the functionalization and size of CNTs[36], thus far the researchers have been unable to control the release profile in a specified manner.

In this study we fabricated a method to engineer a novel CNT based delivery vector functionalized with a degradable PLGA coating. Through degradation of PLGA, transcription factors are able to be released in a controlled manner and tune cell behavior. Significant advantages to our proposed system include the ability to transfect cells efficiently with the unique needle-like shape of CNTs, reduce cytotoxicity of pristine CNTs through a biocompatible PLGA coating, and program protein release times by controlling the degradation profiles of the PLGA.

## Materials and Methods

### Carbon Nanotubes Carboxylation

CNTs (0.2g) purchased from Nanolab Inc. with an average length of 1-5 µm, diameter of 15±5 nm, and purity higher than 95% were added into a single neck glass flask with 200 ml 70% nitric acid (Fisher). CNTs were homogenously dispersed by sonication for 60 minutes at ambient conditions. A condenser reflux apparatus was equipped to prevent CNTs from drying out. The reaction temperature was set at 120 °C and after 12 hours the reaction was quenched by addition of 200 ml deionized (DI) water. The mixture was filtered, washed with DI water to neutral pH, and dried at 80 °C overnight[37]. 

### PLGA Functionalization

Carboxyl CNTs (50 mg) were homogeneously dispersed in 50 ml dimethylformamide (DMF) (Fisher) by sonication for 2 hours at ambient conditions. A total of 2 ml oxalyl chloride (Acros) was added drop wise under N_2_ and stirred in ice water for 2 hours. This was then stirred for 2 hours at room temperature and transferred into an oil bath at 70 °C to be stirred overnight to remove the excess of oxalyl chloride. PLGA (0.5 g) (75:25) (Lactel) was then added to react with carboxylate CNTs and the mixture was stirred at 100 °C for 5 days. Following this, the mixture was cooled down, filtered, and washed with DMF, ethanol (Decon Labs), and DI water. The PLGA linked CNTs (CNT-PLGA) were the black leftover on the filter paper and were dried at 80 °C overnight and collected [38].

### Protein attachment

CNT-PLGA (100 µg) was dispersed in 0.5 ml of 2-(N-Morpholino)ethanosulfonic acid (pH 5.6) (MES) (Acros) buffer under sonication for 1 hour at ambient conditions. 0.25 ml of 1-(3-Dimethylaminopropyl)-3-ethylcarbodiimide hydrochloride (0.2 mol/L) (EDC) (Acros) and 0.25 ml of *N*-Hydroxysuccinimide (0.1 mol/L) (NHS) (Acros) in MES solution were added to the activated carboxylate groups[39,40]. The mixture was washed with PBS and centrifuged in a 100 kDa molecular weight cutoff centrifugal filter (Millipore) to remove EDC and NHS at 5000 g three times for 30 minutes. Then, 5 µg of protein, either bovine serum albumin (BSA) (Sigma), fluorescent BSA (fBSA) (Sigma), or caspase-3 (CP3) (BD) was added into the CNT-PLGA/PBS solution at 4 °C overnight. The mixture was finally washed and centrifuged in a cutoff filter to remove un-conjugated protein six times at 5000 g for 30 minutes[40]. The protein conjugated CNT-PLGA (CNT-PLGA-CP3/BSA/fBSA) solution was collected and stored at -20 °C.

Pro-Ject protein transfection kit (BD) was used as a reference. Liposome nanoparticles were fabricated through the commercial manual. The actual protein dosages were calculated equally to the amount of CNT-PLGA-CP3 groups.

### Cell culture

Osteosarcoma cells MG-63 (ATCC) were cultured in 90% α-MEM (Lonza) and 10% FBS (Gibco) supplemented with 2 mmol/ml l-glutamine (Sigma). Cells were placed in well plate with a seeding density of 20,000/cm^2^. Testing conjugates were introduced after 4 hours. This time point was set as 0 and cells were then maintained in a humidified incubator at 37°C with 5% CO_2_. 

### MTT assay

Cell viability was assessed using 3-(4,5-Dimethylthiazol-2-yl)-2,5-diphenyltetrazolium bromide (MTT) (Alpha Aesar) calorimetric assay at predetermined time points of 1, 3, 5 and 7 days. In brief, MTT/PBS solution was added into each well (1:5) and incubated at 37 °C for 5 h. This was followed by the removal of medium/MTT solution and addition of 1 ml dimethyl sulfoxide (DMSO) (Fisher). The resulting solution was diluted by DMSO in a ratio of 4:1 and the absorbance was read at 550 nm using Tecan SpectroFluo Plus reader. Cell viability was determined as the equation: 

$$\text{Cell Viability=}\frac{\text{Abs550nm of Treated Sample}}{\text{Abs550nm of Control}}\times 100\%$$

### Spectra measurement

XRD patterns were carried out on a Rigaku MiniFlex II with a scanning speed of 0.2 degrees per minute. UV was measured on a Jasco UV 60 UV-Vis spectrometer from 250 to 500 nm. Fourier Transform Infra-Red (FTIR) spectrum was measured on a Nicolet 6700 FT-IR spectrometer from 3600 to 400 cm^-1^. 

### Immunofluorescent images

To remain sterile, cover slips were rinsed in 70% ethanol for 10 minutes and washed with PBS. Cells were seeded onto cover slips and allowed 12 hours to expand on glass slides. The addition of 0.05, 0.1, 0.5, 1 and 3 µg/ml CNT-PLGA-fBSA conjugate was added and incubated for 4 hours. Then samples were washed with PBS, fixed with formalin (Sigma), and dried. Fluorescent images were taken with a Nikon Eclipse 60i microscope system and all images were analyzed with Nikon Elements NX4 software.

### TEM

Cells were seeded onto 40 mesh carbon grids for 12 hours with a density of 20,000 cells/cm^2^ followed by an addition of 3 µg/ml CNT-PLGA-BSA conjugate solution and incubated for 4 hours. Samples were then washed with PBS, fixed with formalin, and dehydrated by a series of ethanol treatments[41]. TEM images of the cell penetration of the CNT-PLGA-BSA conjugate were taken with a Hitachi H8000 TEM. 

### Statistical analysis

Five samples were analyzed at each condition. Data in graphs represents the mean ± standard deviation (SD). Comparison between the two means was determined using the Tukey method and statistical significance was defined as p ≤ 0.05.

## Results and Discussion

### PLGA functionalization

CNTs have been used previously to deliver biological materials across the cell membrane including proteins, DNA, and RNA. Developing the proper surface functionalization for CNTs is the most critical step for a desired application. The two major types of functionalization for CNTs are non-covalent and covalent bonds[42]. Non-covalent binding is based on π-π stacking between the CNTs and aromatic groups from the linkers[43]. Liu et al. showed previously that DNA could be immobilized on pyrene derivatives of functionalized CNTs[44]. Other than these pyrene derivatives, single stranded DNA can also be used to immobilize CNTs[45], however DNA can be cleaved by serum, suggesting non-covalent reactions may not be stable in some cases[46]. For covalent binding, reactive groups are usually formed by various oxidation methods[37,47] which allow for further modifications to enhance polymer[38], protein[48] and DNA[33] attachment. Functionalized CNTs can also be easily dispersed in water, which allows for forming supermolecular bioconjugates such as PLGA and PEG. PLGA is a FDA approved polymer for clinic use with the unique ability to control degradation rate, which allows us to tune the drug release profile. PEG is a commercially manufactured clinical material which has been previously conjugated to CNTs in order to deliver siRNA through the instant cleavage of the S-S bond. PEG has a single functional group and lower average molecular weight when compared to PLGA. Further modifications are thus required to attach active biological molecules to PEG and control the release profile. To this end, a novel CNT-PLGA based drug delivery system has several advantages including a high transfection rate, reduced toxicity, and a highly controlled drug release profile.

**Figure 1:**
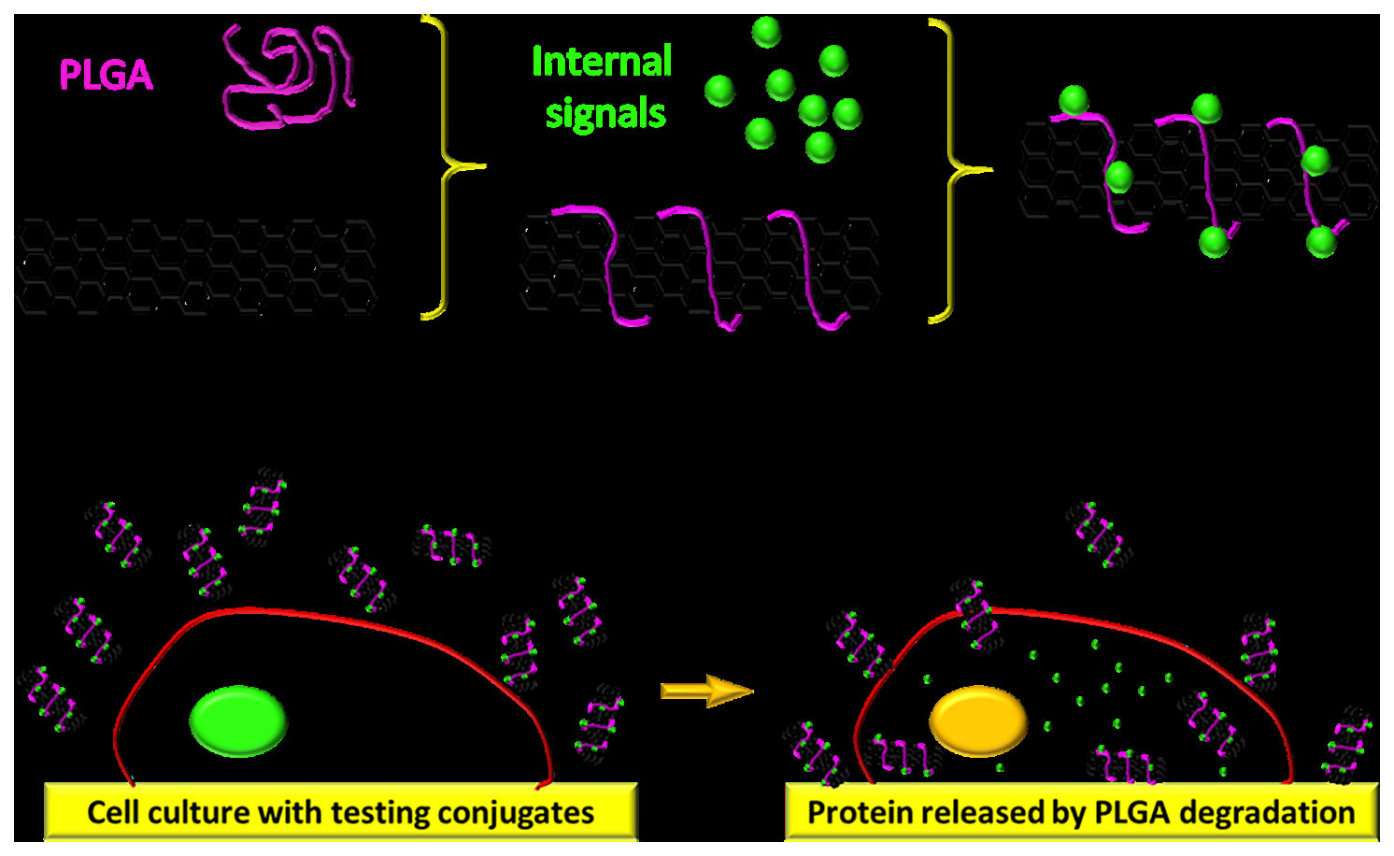
Schematic representation of CNT-PLGA conjugate fabrication and intercellular delivery. CNTs are carboxylated, coated with PLGA, and functionalized with caspase-3. The conjugates are able to penetrate into MG-63 cells and release caspase-3 through the degradation of PLGA.

**Figure 2:**
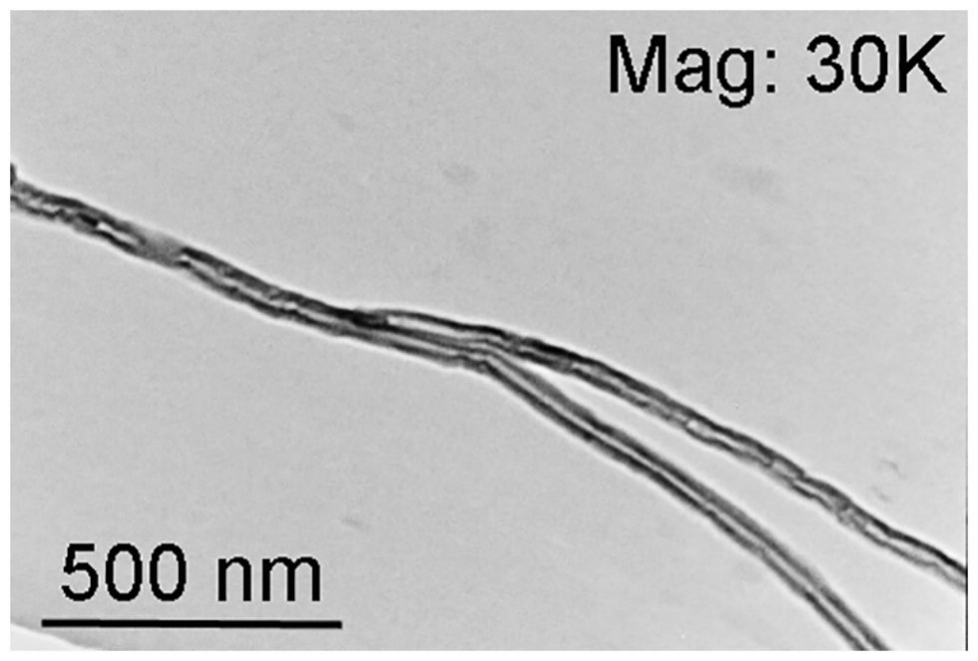
Pristine carbon nanotubes prior to conjugation. TEM image of CNTs. The results show that CNTs have no outer coating prior to PLGA and protein conjugation.

In this study, a novel CNT based drug delivery system as depicted in Figure 1 was developed. In general, pristine CNTs as shown in Figure 2 were oxidized in nitric acid to form carboxyl groups. Carboxyl groups of CNTs were then activated by oxalyl chloride and attached to the PLGA[38]. PLGA is able to provide attachment for the desired transcription factor CP3. The CNT-PLGA-CP3 conjugate can be easily dispersed in PBS without aggregation, which otherwise would hinder penetration of the cell membrane. Due to the enzymatic degradation of PLGA, CP3 is able to be released gradually and induce cell apoptosis. The CNT-PLGA-CP3 conjugations are stable for weeks at -20 °C and the delivery profile can be tuned simply by changing the concentrations of PLGA.

**Figure 3:**
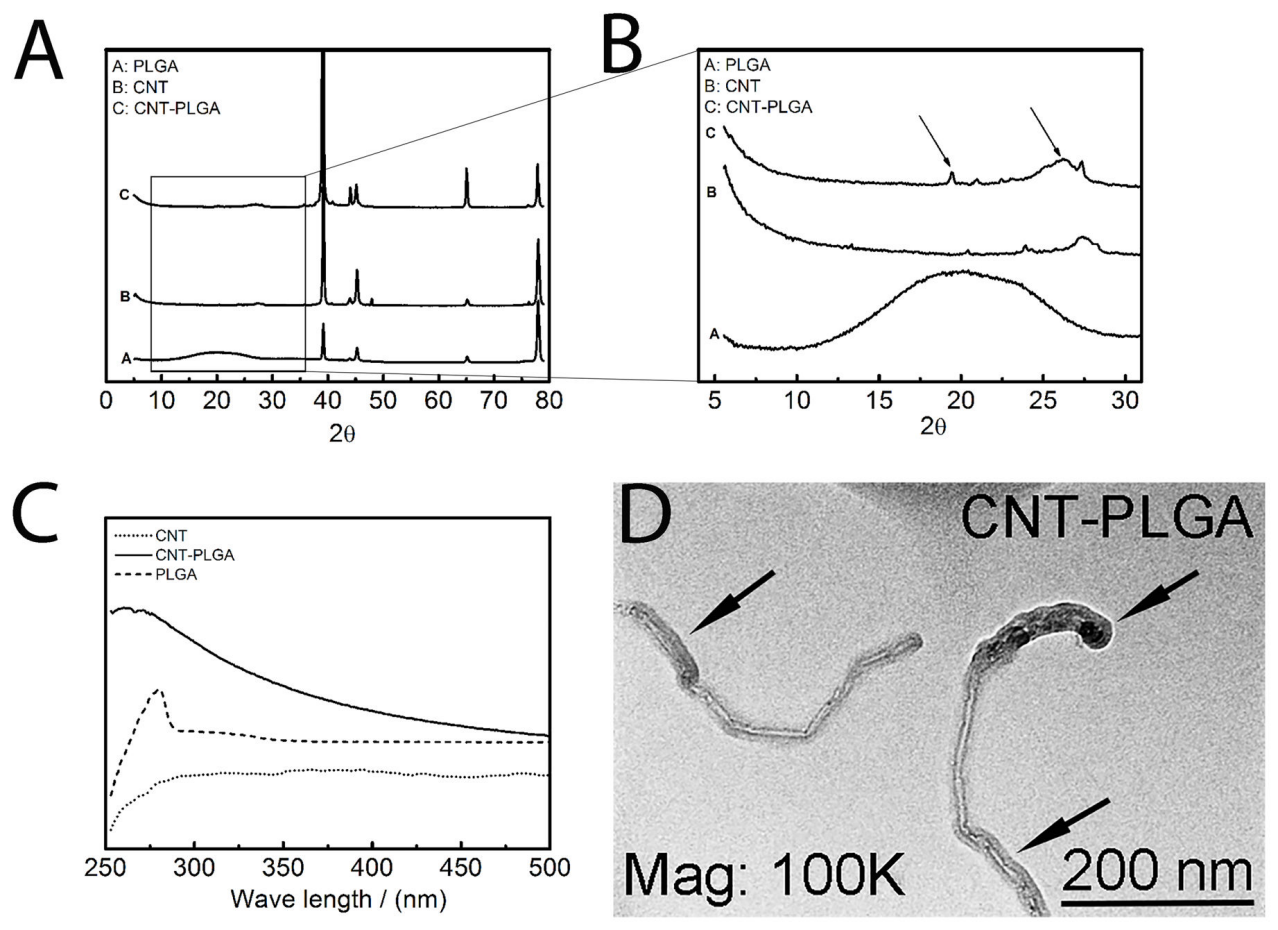
Characterization of CNT-PLGA complex. (A) XRD pattern (B) XRD pattern (C) UV-Vis spectrum (D) TEM image. Results were able to show that PLGA is successfully conjugated to CNTs.

**Figure 4:**
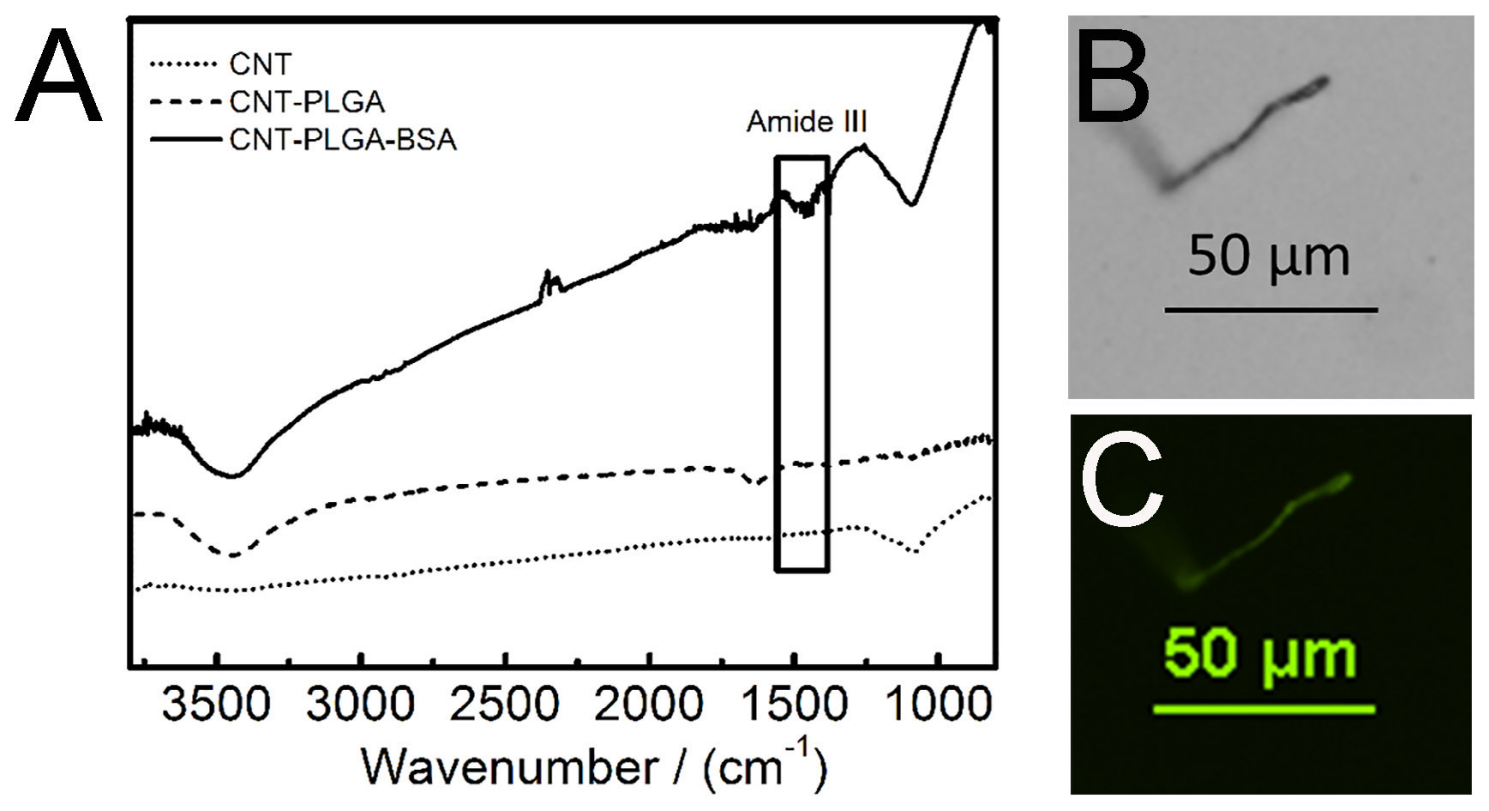
Protein linkage to CNT-PLGA conjugates. (A) Infra-red spectrum (B) light microscopy of CNT-PLGA-BSA (C) fluorescent images of CNT-PLGA-fBSA. Results show that protein has been successfully attached to CNT-PLGA complex, forming CNT-PLGA-BSA conjugates.

 XRD, UV, IR and TEM were used to investigate the CNT-PLGA complex. In Figure 3A, all XRD patterns were roughly the same in the region larger than 35 degrees which represents the crystal structure of the C-H bond, *sp* ^2^ hybrid C-C bond, and C-O bond [49]. However, PLGA patterns showed a peak, which denoted the amorphous region of PLGA polymers from 10-25 degrees as shown in Figure 3B. This peak can be determined as a characteristic of PLGA, which CNTs alone do not possess. A small peak at 22.7 degrees for amorphous PLGA and a peak at 24.1 degrees for CNTs were observed, indicating PLGA attachment to CNTs. However, this peak was not as pronounced as observed in pattern A. We believe only small amounts of PLGA were able to bind to CNTs resulting in a decreased signal. CNTs have a simple flat UV absorption and start to decrease from 280 nm as shown in Figure 3C. PLGA didn’t show a similar trend, instead it showed a strong absorption at 260 nm similar to other studies[50]. CNT-PLGA showed absorption at 260 nm indicating PLGA and a flat decreasing trend thereafter, indicating CNTs were present. This finding showed a similar trend to CNT-Paclitaxel[34]. Through FTIR spectra CNTs showed a typical *sp* ^2^ hybrid C-C stretching at 1200 cm^-1^, while no O-H stretching was observed at 3400 cm^-1^ as shown in Figure 4A. On the contrary, O-H stretching and C=O stretching at 1650 cm^-1^ were observed in CNT-PLGA. This denoted a successful attachment of PLGA to the CNTs. In addition to FTIR, TEM was used to observe the PLGA linkage to CNTs. Pristine multi-wall CNTs in Figure 2 with no PLGA functionalization was compared to the dark PLGA layer clearly shown coated over the CNTs with a depth of several nanometers in Figure 3D.

### Protein attachment

DNA and RNA are the genetic material generally transported by functionalized CNTs. In 2004, Pantarotto discovered CNTs as a tool to transport DNA plasmids[25]. Further research found DNA was protected by CNTs during cellular uptake[51]. In addition, Zhang et al. successfully transported telomerase inhibited small interference RNA into tumor cells and suppressed their growth[52]. However, researchers generally used protein fictionalization for stabilizing [53], labeling[54] and separating[55] CNTs rather than transporting functional protein to tune cellular behavior. Here, we were able to conjugate protein to the CNT-PLGA complex. 

Protein typically has a UV absorption at 280 nm due to *sp* ^2^ hybrid C-C in Phe, Tyr, and Trp which is a similar structure to CNTs. Protein also has a similar amorphous region in XRD pattern as most polymers. Therefore, XRD and UV are not appropriate methods to confirm protein attachment so we used FTIR and fluorescently tagged BSA to confirm the attachment of protein. As shown in Figure 4A, CNT-PLGA-BSA showed a peak around 1400 cm^-1^ indicating amide III belt, which is the typical peak of BSA. fBSA was then used to attach to CNT-PLGA and in Figure 4B and 4C, a single CNT-PLGA attached to fBSA was observed through immunofluorescence indicating successful conjugation. CNTs are generally auto fluorescent over 800 nm [56] but no fluorescent signal was observed at those wavelengths confirming the protein attachment.

### Cellular transfection by CNT-PLGA-fBSA conjugates

**Figure 5:**
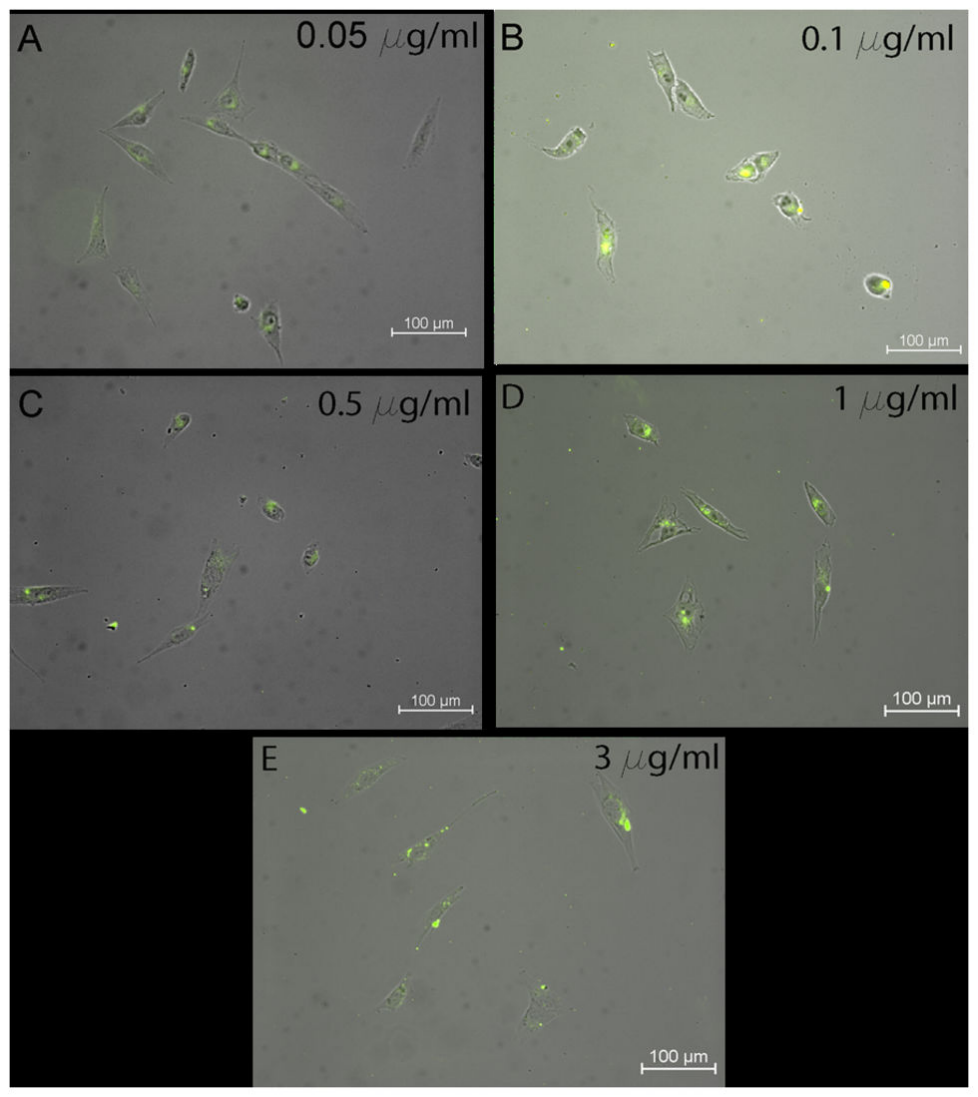
Optic and fluorescent combined images of CNT-PLGA-fBSA delivery into MG-63 osteosarcoma cells. The dosages with osteosarcoma cells were 0.05 μg/ml, 0.1 μg/ml, 0.5 μg/ml, and 1 μg/ml and 3 μg/ml, respectively. The results showed that CNT-PLGA-fBSA could easily penetrate into cells and transduce osteosarcoma cells. All images are 20x magnification.

**Figure 6:**
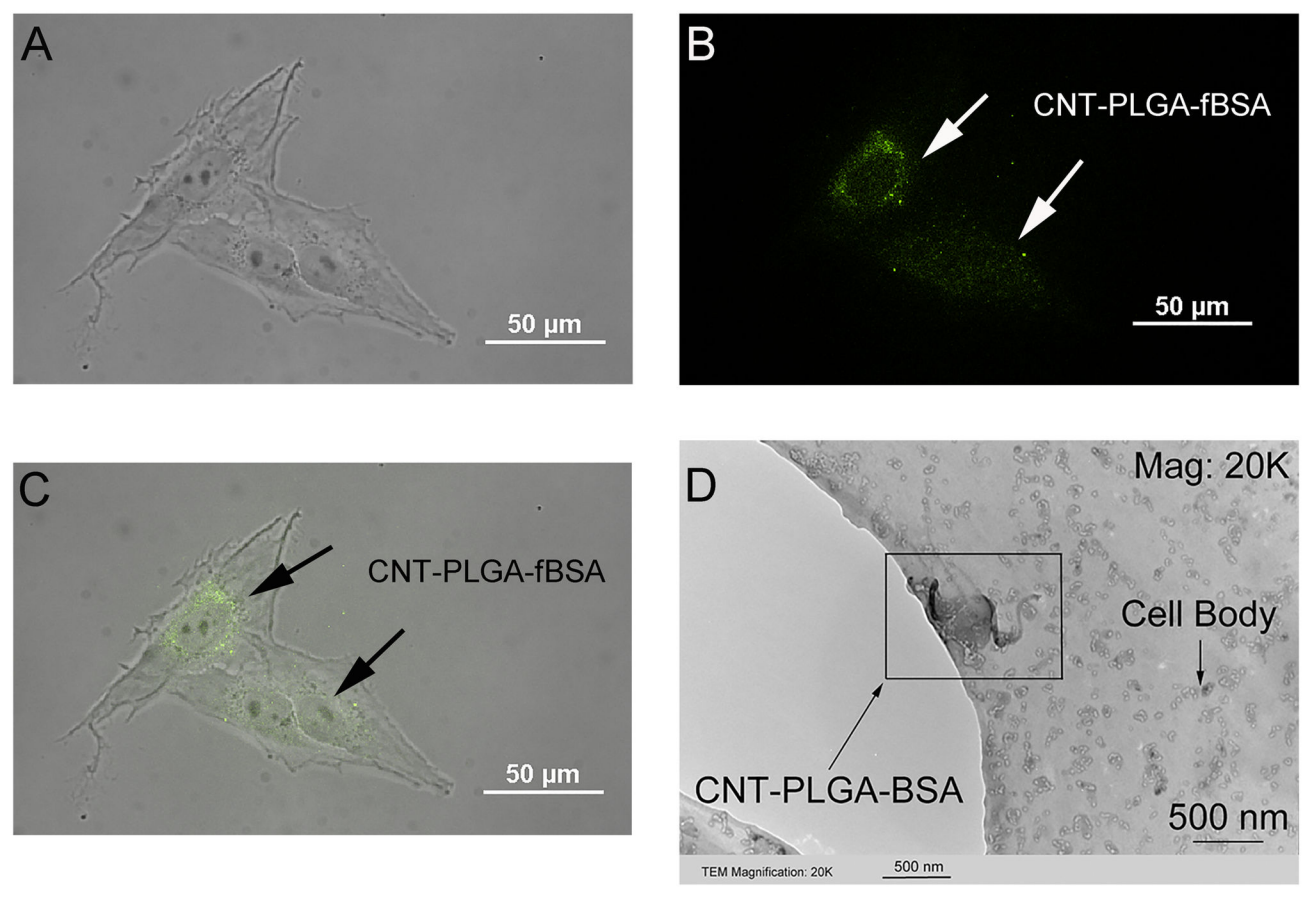
Penetration of CNT-PLGA-proteins into the cells. Determination of CNTs delivered into osteosarcoma cells. Dosage is 3 μg/ml. (A) Osteosarcoma cells cultured with CNT-PLGA-fBSA (B) Image of fluorescent signal within osteosarcoma cells (C) Optic and fluorescent combined image of CNT-PLGA-fBSA image (D) TEM image of a single CNT-PLGA-BSA penetrating the cell body.

The ideal vector to deliver biological material should have two basic characteristics being high efficiency and safety. In terms of efficiency, we investigated the cellular uptake ability of CNT-PLGA-fBSA and CNTs were found to have the ability to penetrate cells due to their nano-sized diameter and small radius to volume ratio[57]. In demonstrating the high efficiency to penetrate cells, we tested the transfection ability of CNT-PLGA-fBSA conjugations in different dosages. In order to prevent non-specific background signals, cells were carefully washed to remove any non-penetrated CNT-PLGA-fBSA conjugates. We were able to observe fluorescent signals in cells as shown in Figure 5. The CNT-PLGA-fBSA conjugates showed a pronounced ability to penetrate cells with a transfection rate close to 100% at all conducted concentrations, ranging from 0.05 to 3 μg/ml. The high magnification image of Figure 6A, 6B, and 6C are able to show higher resolutions around the nuclei which shows higher fluorescent signal, coinciding with the location of where large amounts of ribosomes reside. This positioning of the CNT-PLGA-fBSA conjugates facilitates RNA and transcription factor delivery to the cells. TEM was also used to confirm cell penetration as shown in Figure 6D, where clearly there is a CNT-PLGA-BSA conjugate penetrating into the cytosol, demonstrating the ability of CNTs to transport material across the membrane. We have successfully achieved a transfection rate higher than the 30% or 40% attained by polymers and liposome nanoparticles indicating the promise in non-viral gene delivery vectors.

### The efficacy of CNT-PLGA-CP3 in tuning apoptosis

 Commonly used drugs for cancer therapies are alkylating agents, anti-metabolites, plant alkaloid and terpenoids, topoisomerase inhibitors, and cytotoxic antibiotics. These chemical agents generally present side effects which not only kill cancer cells, but also induce necrosis of healthy tissue. In order to prevent or limit these side effects, we have chosen CP3 as a potential candidate. CP3 is an enzyme that is highly involved in the cell apoptosis pathway. We used MTT assays to test cell viability as a standard to measure the ability of CNT-PLGA-CP3 to release CP3 to cells. The working concentration is set at low levels in order to eliminate cell necrosis induced by large amounts of CNTs. Researchers have demonstrated functionalized CNTs were found to be less toxic than pristine CNTs[58,59], and pristine CNTs in low concentrations have been shown to exhibit acceptable toxicity levels[60]. So cell death experienced in this experiment is attributed to the contribution of CP3 delivered to cells inducing apoptosis. 

**Figure 7:**
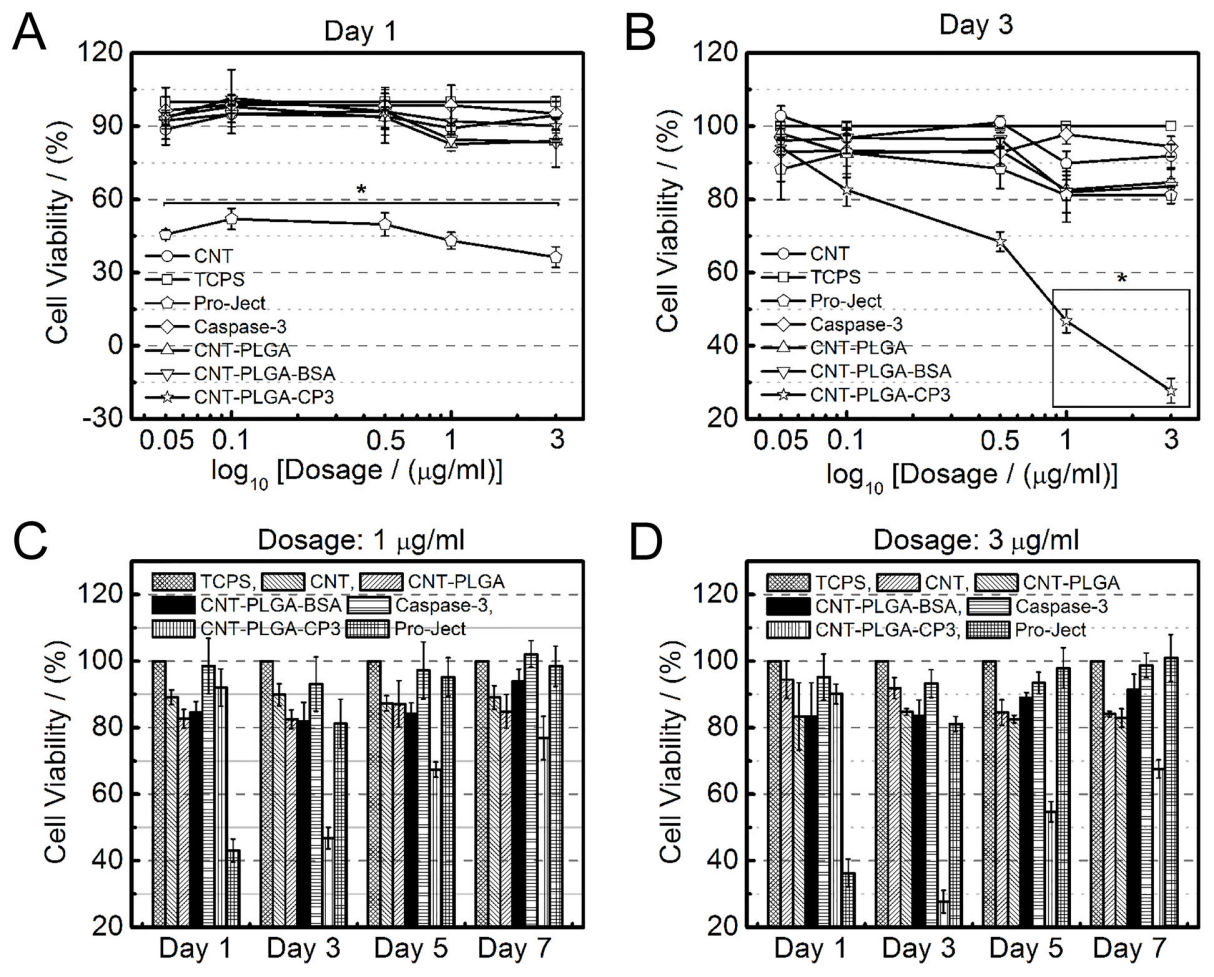
Carbon nanotubes have the ability to penetrate cells and release caspase-3. Cell viability on day 1 (A) and day 3 (B) of MG-63 cells under exposure to differing dosages of CNTs, CNT-PLGA, CNT-PLGA-BSA, caspase-3, Pro-Ject and CNT-PLGA-CP3 treatment. * means significant difference of cell viability under CNT-PLGA-CP3 cojugate exposure and other groups of same dosage.

Pro-Ject is a commercially available liposome generally used for protein delivery which we used as a positive control in comparison to our CNT-PLGA-CP3 conjugation. As shown in Figure 7A, no significant differences were observed in CNT-PLGA-CP3 groups on day 1, however, Pro-Ject groups showed significant difference in comparison. We attribute this to the fact that the degradation of PLGA hasn’t fully allowed the release of significant amounts of CP3[61]. We went on to further demonstrate that limited protein was able to be released in the first 2 days of degradation with an *in vitro* release profile (Figure S1 and S2 in File S1). Pro-Ject liposomes released material with minimal differences in dosage on Day 1. We hypothesize that the average amount of CP3 delivered was significant enough to induce apoptosis even at minimal concentrations. Interestingly, the cell viability of all Pro-Ject liposome samples average at approximately 50%. Considering the CP3 was enough to induce apoptosis, the efficiency of Pro-Ject liposome groups was close to 50%.

Figure 7B shows the cell viability of CNT-PLGA-CP3 treated samples were significantly smaller compared to positive and negative controls on Day 3. Pro-Ject liposome groups treated samples showed minimal differences in this case, indicating there was little to no consistency of material delivery over long periods. Conjugate dosage played an important role in inducing cell apoptosis with no significant differences observed between CNT-PLGA-CP3 in low dosages and controls while CNT-PLGA-CP3 conjugates in high concentration exhibited a significantly low cell viability compared to other controls. We believe the amount of transcription factor reacting with the CNT-PLGA complex is the same but the amount of CP3 released was highly dependent on the conjugate dosage. 

We also conducted long-term cell viability tests at two high dosages with no additional treatments throughout the experiment. The results shown in Figure 7C and 7D show that cell viability was still suppressed by treating 1 µg/ml CNT-PLGA-CP3 on Day 5 and 3 µg/ml CNT-PLGA-CP3 on Day 7, respectively. Note that cancer cells are able to recover and become fully confluent under Pro-Ject liposome groups between Day 1 and Day 3. We believe CP3 is superior due to the gradual release profile.

## Conclusions

We were able to successfully fabricate a CNT-PLGA system, which is able to deliver biological material including genes, transcriptional factors, and signal molecules into cells. This system has shown a high transfection rate and a reliable, time dependent release profile. Yet another advantage to this system is that the releasing profile can be tuned simply by controlling the molecular weight and ratio of PLGA (Figures S3 and S4 in File S1). In all aspects, this CNT-PLGA-CP3 conjugation is a highly efficient and promising drug delivery system with possible future applications in developing scaffolds for bone tissue engineering.

## Supporting Information

File S1 **File includes Figures S1-S4.**
**Figure S1:** Calibration curve of BSA concentration vs UV aborption at 280 nm.
**Figure S2:** BSA release fraction from the CNT-PLGA-BSA conjugates at predetermined time points. CNT-PLGA-BSA dispersed in PBS was incubated in a water bath (37°C).
**Figure S3:** Calibration curve of resveratrol concentration versus UV aborption at 327 nm.
**Figure S4:** Resveratrol release fraction from the PLGA nanoparticles. PLGA nanoparticles were made from PLGA with differing ratios of PLA to PGA and molecular weight (5,000-16,000/50:50, 16,000-29,000/50:50 and 75,000-100,000/75:25).
(DOCX)
