# Correction: PLGA-Carbon Nanotube Conjugates for Intercellular Delivery of Caspase-3 into Osteosarcoma Cells

**DOI:** 10.1371/annotation/33c838f9-dc56-402f-bc29-f526c9472ec2

**Published:** 2014-01-03

**Authors:** Qingsu Cheng, Marc-Olivier Blais, Greg M. Harris, Ehsan Jabbarzadeh

The name of the third author is incorrect. The correct name is: Greg M. Harris.

The correct Citation is: Cheng Q, Blais M-O, Harris GM, Jabbarzadeh E (2013) PLGA-Carbon Nanotube Conjugates for Intercellular Delivery of Caspase-3 into Osteosarcoma Cells. PLoS ONE 8(12): e81947. doi:10.1371/journal.pone.0081947.

The correct abbreviation of the third author's name in the Author Contributions Statement is: GMH.

