# Supplementary material for: PLGA-Carbon Nanotube Conjugates for Intercellular Delivery of Caspase-3 into Osteosarcoma Cells
Source: PLoS One. 2013 Dec 3;8(12):e81947. doi: 10.1371/journal.pone.0081947 (PMC3849501; doi:10.1371/journal.pone.0081947)
Supplement: File S1 — File includes Figures S1-S4. Figure S1: Calibration curve of BSA concentration vs UV aborption at 280 nm. Figure S2: BSA release fraction from the CNT-PLGA-BSA conjugates at predetermined time points. CNT-PLGA-BSA dispersed in PBS was incubated in a water bath (37°C). Figure S3: Calibration curve of resveratrol concentration versus UV aborption at 327 nm. Figure S4: Resveratrol release fraction from the PLGA nanoparticles. PLGA nanoparticles were made from PLGA with differing ratios of PLA to PGA and molecular weight (5,000-16,000/50:50, 16,000-29,000/50:50 and 75,000-100,000/75:25). (DOCX) [file pone.0081947.s001.docx]

**PLGA-Carbon Nanotube Conjugates for Intercellular Delivery of Caspase-3 into Osteosarcoma Cells**

**Supplementary File S1**

Qingsu Cheng^a^, Marc-Olivier Blais^b^, Greg Harris^b^, and Ehsan Jabbarzadeh^a,b,c *^

^a^Department of Biomedical Engineering, University of South Carolina, Columbia, SC, United States

^b^Department of Chemical Engineering, University of South Carolina, Columbia, SC, United States

^c^Department of Orthopaedic Surgery, University of South Carolina, Columbia, SC, United States

* Corresponding author

**Ehsan Jabbarzadeh Ph.D.**

Assistant Professor of Chemical Engineering

Assistant Professor of Biomedical Engineering

Assistant Professor of Orthopaedic Surgery

University of South Carolina

Columbia, SC 29208

Ph: (803) 777-3297

Fax: (803) 777-8265

Email: [ehsan@sc.edu](mailto:ehsan@sc.edu)

**Material and Method**

*BSA release profile*

CNT-PLGA (100 µg) was dispersed in 0.5 ml of 2-(N-Morpholino)ethanosulfonic acid (pH 5.6) (MES) (Acros) buffer under sonication for 1 hour at room temperature. 0.25 ml of 1-(3-Dimethylaminopropyl)-3-ethylcarbodiimide hydrochloride (0.2 mol/L) (EDC) (Acros) and 0.25 ml *N*-Hydroxysuccinimide (0.1 mol/L) (NHS) (Acros) in MES solution were added to the activated carboxylate groups [[1](#_ENREF_1),[2](#_ENREF_2)]. The mixture was washed with PBS and centrifuged in a 100 kDa molecular weight cutoff centrifugal filter (Millipore) to remove EDC and NHS at 5,000 g three times for 30 minutes. Then, 200 µg of BSA was added to the CNT-PLGA/PBS solution at 4 ^o^C overnight. Lastly, the mixture was washed and centrifuged in a cutoff filter to remove un-conjugated BSA six times at 5,000 g for 30 minutes [[2](#_ENREF_2)]. The removed filtered solution was collected each run to determine amount of loaded BSA.

*Nano-particle preparation*

In general, 100 mg of PLGA (Lactel) in differing ratios of PLA to PGA and molecular weight (5,000-16,000/50:50, 16,000-29,000/50:50 and 75,000-100,000/75:25) along with 10 mg of resveratrol (Sigma) were dissolved in 5 mL acetone (Sigma). The mixture was then slowly injected into 100 mL 0.25 mol/mL MES solution (pH 5.0) containing 2.5% PVA (Sigma) at a rate of 1 mL/min [[1](#_ENREF_1),[2](#_ENREF_2)]. After 24 hours of moderate stirring to remove traces of acetone, the nanoparticle solution was centrifuged 6 times to remove the non-incorporated resveratrol. The supernatants were collected to determine the loaded resveratrol. The collected nanoparticles were lyophilized and stored in a desiccator.

*Determination of release profile*

The CNT-PLGA-BSA conjugates were then dispersed in 1 mL PBS by sonication (5 min) and incubated in a water bath (37 ^o^C). The entire conjugate solution was centrifuged in a 100 kDa molecular weight cutoff centrifugal filter (Millipore) to remove the released BSA solution at each predetermined time point. The concentration of the released BSA was determined with a Jasco UV 60 UV-Vis spectrometer at 280 nm with a pre-made calibration curve (Supplementary Figure 1).

The nanoparticles (1mg) were dispersed in 1 mL PBS by sonication (5min) in an EP tube and incubated in a water bath (37 ^o^C). The nanoparticle suspension was centrifuged to collect the supernatant at each predetermined time point. The concentration of released resveratrol was determined with a Jasco UV 60 UV-Vis spectrometer at 327 nm with a pre-made calibration curve (Supplementary Figure 3).

**Result and Discussion**

In order to detect the BSA release with a UV-Vis spectrometer, we increased the amount of BSA (200 µg compared to 5 µg of caspase-3) reacting with the CNT-PLGA complex. This increased amount of BSA resulted in BSA released at detectable levels through UV-Vis spectrometer. The protein loaded onto 100 µg of CNT-PLGA was 119±6.73 µg per 200 µg. We observed a gradual release of BSA through the time points in the 10-day trial (Supplementary Figure 2). Throughout the first two days we saw only a slight release of BSA while more than 5 µg of BSA was released after Day 7. This is a possible explanation for the CNT-PLGA-CP3 conjugates showing limited inhibitory effects on Day 1 while the CNT-PLGA-CP3 conjugates showed significantly higher inhibitory effects after Day 3. With only 5 µg of CP3 in the starting reaction, this may help explain the inhibitory effects on cell growth diminishing after Day 7. We also observed an increase in BSA released from Day 8 to Day 10. This is because the large molecular weight PLGA was hydrolyzed to a small fraction, which leads to an increase in protein release. Due to the fact that the CNT-PLGA-CP3 conjugates face a more complicated enzymatic environment, it is extremely difficult to quantitatively monitor the release profile *in situ*. However, this release profile data in combination with our cytotoxicity data give us confidence in the reliability of our results.

In determining the release profiles of the CNT-PLGA with differing PLGA to PGA and molecular weights, we simplified the experiment using PLGA nanoparticles as an alternative. We observed in general that resveratrol was gradually released from all three groups of PLGA nanoparticles (Supplementary Figure 4). Note that because the resveratrol was not covalently bound to the PLGA, the release fraction of resveratrol is higher than that of BSA, which is covalently bound to the CNT-PLGA complex. The amount of resveratrol loaded on to the PLGA nanoparticles are 38%, 36%, and 40%. Interestingly, the 3 groups of PLGA nanoparticles showed different trends of release profiles with the smaller molecular weight PLGA nanoparticles releasing more resveratrol than the higher molecular weight counterparts. Considering the method to covalently bind the protein to the CNT-PLGA, the conjugates would require more time for the same fraction of BSA released.

Figure S1

Figure S2

Figure S3

Figure S4

**References**

1. McCarron PA, Marouf WM, Donnelly RF, Scott C (2008) Enhanced surface attachment of protein-type targeting ligands to poly(lactide-co-glycolide) nanoparticles using variable expression of polymeric acid functionality. Journal of Biomedical Materials Research Part A 87A: 873-884.

2. Liu Z, Tabakman SM, Chen Z, Dai HJ (2009) Preparation of carbon nanotube bioconjugates for biomedical applications. Nature Protocols 4: 1372-1382.
